# WuYeLuGen Granule Attenuates Bleomycin‐Induced Pulmonary Fibrosis in Rats by Inhibiting the TGF‐β1/Smad Signaling Pathway and Epithelial–Mesenchymal Transition

**DOI:** 10.1155/carj/8103859

**Published:** 2026-02-23

**Authors:** Jia-Wei Zeng, Li Lin, Yan-Jun Duan

**Affiliations:** ^1^ Department of Physiology, College of Basic Medical Sciences, Hubei Shizhen Laboratory, Hubei University of Chinese Medicine, Wuhan, 430065, China, hbtcm.edu.cn; ^2^ Department of Anatomy Teaching and Research, College of Basic Medical Sciences, Hubei University of Chinese Medicine, Wuhan, 430065, China, hbtcm.edu.cn

**Keywords:** epithelial–mesenchymal transition, pulmonary fibrosis, TGF-β1/Smad pathway, WuYeLuGen Granule

## Abstract

**Background:**

Pulmonary fibrosis is a chronic disease characterized by progressive interstitial lung changes affecting alveolar epithelial cells and pulmonary vessels. Following COVID‐19, it has emerged as a significant sequela in severe cases, often with a poor prognosis. WuYeLuGen (WYLG) Granule, derived by Xue’s Wuye Lugen Granule, exerts effects of replenishing qi, nourishing yin, clearing heat, and resolving dampness. While clinical and experimental studies provide evidence to support WYLG’s efficacy against early‐stage pulmonary fibrosis, its underlying mechanisms remain incompletely understood.

**Methods:**

Active components of WYLG were identified using LC‐MS/MS. CCK8 assays were performed to determine the optimal concentrations of WYLG‐containing serum and TGF‐β1. WYLG granules were administered to bleomycin (BLM)‐induced rats and WYLG‐containing serum was applied to TGF‐β1‐stimulated rat pulmonary fibroblasts (RPFs). Hematoxylin‐Eosin (HE) and Masson staining were used to assess the protective effects of WYLG on rat lung tissues, while enzyme‐linked immunosorbent assay (ELISA) was employed to evaluate lung inflammation. Flow cytometry analyzed RPF cell proliferation, scratch assays examined cell migration, and Western blot detected the expression of fibrotic and pathway‐related proteins. Immunofluorescence was used to confirm the efficacy of WYLG in reducing RPF cell fibrosis.

**Results:**

LC‐MS/MS identified 18 active components in WYLG, primarily derived from *Salvia miltiorrhiza* and *Astragalus membranaceus*. The optimal concentration for TGF‐β1‐induced RPF stimulation was 10 ng/mL, and the optimal concentration of WYLG‐containing serum was 10%. In BLM‐induced rats, WYLG granules significantly alleviated pulmonary fibrosis, reduced inflammatory cell infiltration and collagen deposition, downregulated IL‐6 and α‐SMA levels, and upregulated E‐cadherin expression. Mechanistically, WYLG treatment decreased the levels of TGF‐β1 and p‐Smad2/Smad2, while increasing Smad7 levels in rat lung tissue. In TGF‐β1‐stimulated RPF, WYLG‐containing serum normalized cell proliferation, inhibited cell migration, reduced collagen I and α‐SMA expression, and increased E‐cadherin expression. Consistent with animal experiments, WYLG‐containing serum also downregulated TGF‐β1 and p‐Smad2/Smad2 levels in RPFs. Additionally, the TGF‐β1/Smad pathway agonist SRI‐011381 reversed the inhibitory effects of WYLG on RPF fibrosis, further confirming that WYLG exerts its antifibrotic effect through the TGF‐β1/Smad pathway.

**Conclusions:**

WYLG markedly alleviates pulmonary fibrosis both in vivo and in vitro by inhibiting the TGF‐β1/Smad signaling pathway and regulating epithelial‐to‐mesenchymal transition, highlighting its potential as a therapeutic agent for progressive pulmonary fibrosis.

## 1. Introduction

Pulmonary fibrosis is a diffuse interstitial lung disease characterized by chronic progressive changes in the pulmonary interstitium, involving excessive deposition of extracellular matrix (ECM) components and abnormal cellular responses. It frequently affects alveolar epithelial cells and pulmonary vasculature [1]. In recent years, the prevalence of pulmonary fibrosis has risen markedly, with an average survival time post‐diagnosis estimated at only 3–5 years [2]. Following the outbreak of COVID‐19, pulmonary fibrosis has emerged as one of the most severe sequelae among patients with severe or critical illness, and prognosis remains grim [3]. Consequently, enhancing research efforts aimed at preventing and treating pulmonary fibrosis is crucial for reducing its incidence and fostering better life outcomes for patients.

The mechanisms of pulmonary fibrosis remain incompletely understood, with several mechanistic hypotheses proposed. Among these, the epithelial–mesenchymal transition (EMT) of alveolar epithelial cells is one of the most important hypotheses [4]. EMT is a crucial factor in the transformation of alveolar epithelial cells into mesenchymal cells, and modern medical research also recognizes that EMT occurs in the development of fibrosis in various organs [5, 6]. This process is related to the regulation of multiple signaling pathways, including Ras, Rho, Src, Col I, and Smads. This transition is marked by the loss of expression of the cell adhesion molecule E‐cadherin and the upregulation of cytoskeletal protein α‐SMA, resulting in a fibroblast‐like morphology [7]. Nuclear factor kappa B (NF‐κB) is a key mediator of EMT. It promotes the transcription of various inflammatory cytokines such as tumor necrosis factor alpha (TNF‐α), interleukins (IL), and transforming growth factor beta 1 (TGF‐β1). These factors are critically involved in the pathogenesis of pulmonary fibrosis, with TGF‐β1 playing a central role. Furthermore, the pathological process confirms that pulmonary fibrosis is intricately linked with inflammation [8]. As a multifunctional cytokine, TGF‐β1 is central to the pathogenesis of organ fibrosis, with the TGF‐β1/Smad signaling pathway playing a decisive role in the progression of pulmonary fibrosis [9–11]. In clinical treatment, most interventions occur after pulmonary fibrosis has already progressed, making treatment relatively difficult. Current medications can only slow disease progression and extend patient survival rather than achieve a cure [12]. Therefore, investigating early intervention strategies, particularly through traditional Chinese medicine (TCM), is of significant importance for potentially reversing the disease process and improving patient outcomes.

“Preventing diseases before they occur” is a fundamental concept in the theory of disease prevention within TCM. It serves as the theoretical foundation for the approach of “treating diseases before they manifest.” This principle involves anticipating the developmental trajectory of diseases at an early stage and implementing appropriate measures to control them, thereby facilitating improvement or even recovery [13]. When the lungs are subjected to stimulation, recurrent lung inflammation and persistent alveolar injury can trigger protective self‐repair mechanisms within the lungs, ultimately leading to pulmonary fibrosis. Therefore, early intervention with TCM is crucial to prevent the progression to pulmonary fibrosis during its initial stages [14].

According to TCM theory, the core pathogenesis of early‐stage pulmonary fibrosis is characterized by qi and yin deficiency, accompanied by blood stasis [15]. The WuYeLuGen (WYLG) Granule, developed from Xue’s WYLG Granule by renowned Chinese medicine practitioner Xue Shengbai, is designed to replenish qi and nourish yin while clearing heat and resolving dampness. It also promotes blood circulation to alleviate meridian obstruction. Clinical and experimental studies have demonstrated the therapeutic efficacy of WYLG Granule in facilitating early recovery from pulmonary fibrosis associated with COVID‐19 [16, 17]. Our study aims to investigate the mechanisms underlying the protective effects of WYLG granules against bleomycin (BLM)‐induced early pulmonary fibrosis in rats.

## 2. Materials and Methods

### 2.1. Determination of the Main Chemical Constituents in WYLG Granule

For each group, 1.2 mL of serum was mixed with 4.8 mL of methanol. The mixture was vortexed at 3000 revolutions per minute (r/min) for 90 s, followed by centrifugation at 4°C and 13,000 r/min for 10 min. The supernatant was collected and dried under nitrogen gas, and the resulting residue was redissolved in 150 μL (μL) of 70% methanol. After another vortexing step at 3000 r/min for 90 s and centrifugation at 4°C at 13,000 r/min for 10 min, the supernatant was collected again, and 3 μL of it was injected for liquid chromatography‐tandem mass spectrometry (LC–MS/MS) analysis.

### 2.2. Animal Experiment

#### 2.2.1. Animals

Six‐week‐old male rats (200 ± 10 g) were purchased from Liaoning Changsheng Biotechnology Co., Ltd and acclimatized in the animal facility of Hubei University of Chinese Medicine under controlled conditions, including a temperature range of 24°C, 60% humidity, sufficient ventilation. The rats were adaptively fed for 1 week prior to the experiment. The animal protocol was reviewed and approved by the Ethics Committee of Hubei University of Traditional Chinese Medicine (No. 42010200005322).

#### 2.2.2. Drugs

BLM was purchased from Han Hui Pharmaceuticals Co., Ltd. (Hangzhou, China). WYLG Granule was prepared by the Pharmaceutical Department at Hubei Provincial Hospital of TCM. WYLG consists of Agastache Rugosus (Huo Xiang, 10 g), Eupatorii Herba (Pei Lan, 10 g), Eriobotryae Folium (Pi Pa Ye, 30 g), Benincasa Hispida Cogniaux (Dong Gua Ren, 15 g), Menthae Heplocalycis (Bo He, 10 g), Adenophorae Radix (Nan Sha Shen, 15 g), Radix Astragali Preparata (Zhi Huang Qi, 30g), Stir‐fried Ovate Atractylodes Root (Chao Bai Zhu, 10 g), Salviae Miltiorrhizae (Dan Shen, 15 g), Phragmitis Rhizoma (Lu Gen, 15 g), and Radix Glycyrrhizae (Gan Cao, 10 g). All granules are produced by China Resources Sanjiu Medical & Pharmaceutical Co., Ltd. WYLG Granules were dissolved in 95°C hot water and concentrated to densities of 5 g/mL, 10 g/mL, and 20 g/mL, respectively.

#### 2.2.3. Animal Grouping and Treatment

Rats were randomly divided into five groups (*n* = 10): the control group (CON), the BLM group, the BLM + WYLG‐L group, the BLM + WYLG‐M group, and the BLM + WYLG‐H group. All other rats were anesthetized with an intraperitoneal injection of 1% sodium pentobarbital (40 mg/kg) and then administered a tracheal instillation of 5 mg/kg BLM except for the CON group. Immediately after administration, the rats were inverted to ensure uniform drug distribution in the lungs. Rats in the CON group received an equivalent volume of sterile saline instead of BLM. The animal doses were converted to human equivalent doses based on body surface area, resulting in WYLG doses for rats of 16.28 g/kg (WYLG‐L), 32.55 g/kg (WYLG‐M), and 65.10 g/kg (WYLG‐H) (Nair and Jacob 2016). Starting from the first day of modeling, the WYLG groups received the corresponding WYLG solution at a dose of 0.35 mL/100g daily via gavage. The CON and BLM groups received an equivalent volume of saline through the same method, continuing for 14 days.

#### 2.2.4. Experimental Sampling

On days 1, 3, 5, 7, and 14 post‐inject, rats were randomly selected from the CON, BLM, and WYLG‐H groups and euthanized for lung tissue collection (Combining the results of WYLG in vivo dose‐screening experiments, we confirmed that high‐dose WYLG was the optimal dose). The remaining rats, after completing the drug intervention, were anesthetized with 1% sodium pentobarbital (40 mg/kg) and divided into two batches within each group: one batch had their lungs harvested following perfusion and stored in 4% paraformaldehyde for later analysis. Other batch underwent abdominal aorta blood collection, followed by harvesting of fresh lung tissues which were stored at −80°C for Western blotting.

#### 2.2.5. Histopathological Evaluation

##### 2.2.5.1. HE Staining

Rat lung tissues were preserved with 4% paraformaldehyde, followed by dehydration, paraffin embedding, and transverse sectioning into slices. Hematoxylin and eosin (H&E) staining was performed on these sections prior to light microscopic observation. The degree of lung tissue inflammation and structural destruction was evaluated using a semiquantitative scoring system.

##### 2.2.5.2. Masson Staining

Lung tissues were fixed in 4% paraformaldehyde solution (Solarbio, Beijing, China), followed by dehydration, paraffin embedding, and sectioning. Collagen deposition was evaluated by Masson trichrome staining, and the collagen volume fraction was quantified using ImageJ software.

#### 2.2.6. ELISA

The levels of E‐cadherin, α‐SMA, and IL‐6 in rat lung tissues were measured using ELISA kits (Ruixinbio, Quanzhou, China) in accordance with the manufacturer’s instructions.

#### 2.2.7. Western Blot (WB)

WB was carried out in accordance with the detailed methodology outlined in our prior publication [18]. Primary antibodies (Smad2, AF6449; Smad7, AF5147; Collagen I, AF7001; GAPDH, AF7021; Affinity; p‐Smad2, Cst, 3108T; TGF‐β1, Abcam, Ab215715) were added and incubated overnight at 4°C.

### 2.3. Cell Experiment

#### 2.3.1. Cell Culture

Rat pulmonary fibroblast cells (RPFs) were purchased from Procell Biotechnology Co., Ltd. (Wuhan, China). RPFs were identified by Vimentin immunofluorescence (IF). Cells were cultured in DMEM containing 10% fetal bovine serum and 1% penicillin‐streptomycin at 37°C in a 5% CO_2_ incubator.

#### 2.3.2. Preparation of Drug‐Containing Serum

Twenty SPF‐grade male Wistar rats (200 ± 10 g, 6 weeks old) were purchased and housed as described above. The rats were acclimated for 3 days and then randomly divided into two groups: the CON group and the WYLG group. Rats in the WYLG group were administered a high concentration of WYLG solution at a dose consistent with that used in the animal experiment, while rats in the CON group received an equivalent volume of saline. Administration was performed via gavage for 7 consecutive days. On the 7th day, 2 h after the final gavage, the rats were anesthetized with 1% sodium pentobarbital (40 mg/kg) and subjected to abdominal aorta blood collection. The collected blood was allowed to clot at room temperature for 30 min, followed by centrifugation at 3000 rpm for 15 min to obtain serum. The serum was then heat‐inactivated at 56°C for 30 min and sterilized by filtration through a 0.22‐μm filter membrane to yield drug‐containing serum, which was stored at −20°C for later use.

#### 2.3.3. Cell Grouping and Treatment

Cells were divided into five groups: the CON group (CON), the TGF‐β1‐treated group (TGF‐β1), the WYLG group, the TGF‐β1 inhibitor (LY2109761) group, and the TGF‐β1/Smad pathway agonist (SRI‐011381) group. Subsequently, the WYLG group was treated with the optimal concentration of drug‐containing serum from WYLG‐administered rats, while the remaining four groups received an equivalent concentration and volume of drug‐containing serum from CON‐administered rats. Additionally, the LY2109761 group was treated with 10 μM LY2109761 [19] and the SRI‐011381 group was treated with 10 μM of SRI‐011381 [20, 21] .

#### 2.3.4. Determination of Optimal Drug and Drug‐Containing Serum Concentrations Using CCK‐8 Assay

RPF cells were seeded in 96‐well plates. When the cell density reached 60%–70%, the cells were treated with gradient concentrations of drug‐containing serum and concurrently stimulated with gradient concentrations of TGF‐β1. After 24 h of incubation, the culture medium was removed, and the cells were washed twice with PBS. Subsequently, 100 μL of complete medium containing 10% CCK‐8 solution was added to each well. Following an additional 1‐h incubation, the absorbance at 540 nm was measured using a microplate reader. Cell viability was calculated according to the following formula.

#### 2.3.5. Cell Scratch Assay

RPF cells were seeded in 6‐well plates and cultured to form a confluent monolayer. A straight scratch was created across the monolayer using a 200‐μL pipette tip. After washing with PBS to remove dislodged cells, the cells were placed back into the culture to allow migration into the scratch wound. The healing process was observed and recorded under a microscope immediately (0 h) and 24 h after scratching.

#### 2.3.6. WB

RPFs were harvested by repeatedly scraping them into EP (polypropylene) tubes on ice using RIPA lysis buffer containing phosphatase inhibitors and PMSF. The cells in the EP tubes were then subjected to multiple low‐temperature vortexing steps to ensure thorough interaction between the cells and the reagent, and then at 4°C, and centrifuged at 12000 rpm for 15 min. The protein supernatant was collected using the same method as in the animal experiment. The relevant procedures for protein extraction, SDS‐PAGE, transfer to PVDF membranes, and blocking were repeated as described previously, until the primary antibodies—Smad2 (AF6449, Affinity), Smad7 (AF5147, Affinity), Collagen I (AF7001, Affinity), GAPDH (AF7021, Affinity), p‐Smad2 (3108T, Cell Signaling Technology), and TGF‐β1 (Ab215715, Abcam)—were added. The membranes were then incubated overnight at 4°C.

#### 2.3.7. IF

The cell slides were placed in a six‐well plate, followed by cell passage and drug intervention. The cell slides were washed three times with PBS in the six‐well plate (5 min per wash), followed by fixation with 4% paraformaldehyde (PFA) for 15 min at room temperature. After fixation, the slides were rinsed three times with PBS (3 min per wash) and then permeabilized with 0.5% Triton X‐100 in PBS for 20 min at room temperature. The cell slides were washed three times with PBS (3 min per wash). Excess PBS was blotted gently with absorbent paper, followed by the addition of 5% normal goat serum blocking buffer (prepared in PBST) onto each slide and incubation for blocking at room temperature for 30 min. Without washing, excess blocking solution was aspirated with absorbent paper. A sufficient volume (50 μL per slide) of diluted primary antibody (1:100–200) was added to each slide, which was then transferred to a humidified chamber and incubated overnight at 4°C. For fluorescent secondary antibody incubation, the cell slides were washed three times with PBST (5 min per wash). Excess liquid on the slides was blotted gently with absorbent paper, followed by the addition of diluted fluorescent secondary antibody (1:200 dilution in PBST containing 1% BSA, 50 μL per slide). The slides were incubated at 37°C for 1 h in a humidified chamber, protected from light. After incubation, the slides were washed three times with PBST (5 min per wash). Subsequently, DAPI staining solution (1:1000 dilution, 50 μL per slide) was added, and the slides were incubated in the dark for 5 min to stain cell nuclei. Excess DAPI was removed by washing the slides four times with PBST (5 min per wash). Residual liquid on the slides was blotted gently with absorbent paper, and the slides were mounted with antifade mounting medium. Finally, images were acquired using a fluorescence microscope at room temperature.

### 2.4. Statistical Analysis

All data were expressed as mean ± standard deviation (SD) and statistically analyzed using SPSS Statistics 26.0 software. Differences between two groups were analyzed using Student’s *t*‐test, while those among three or more groups were assessed via one‐way analysis of variance (ANOVA) followed by Tukey’s post hoc test for multiple comparisons.

## 3. Results

### 3.1. The Main Chemical Constituents of WYLG

The LC‐MS/MS technique was used to analyze the extract of WYLG Granule, and total ion chromatography (TIC) was obtained. The mass spectrum peak figures of WYLG Granule in both positive and negative ion modes are shown (Figure 1(a)). We extracted the ion peaks of these metabolites, including caffeic acid, citric acid, calycosin, L‐isoleucine, methylmalonic acid, pantothenic acid, vanillin acid, adenine, dihydroxyphenylpropionic acid, L‐tryptophan, tanshinone IIA, tanshinol, ferulic acid, genistin, azelaic acid, cryptotanshinone, dodecanedioic acid, and linoleic acid. Among these, 6 compounds were derived from Radix Salviae and 12 compounds were from Hedysarum Multijugum Maxim. These compounds are considered the active ingredients of WYLG Granule (Table 1).

Figure 1The main chemical constituents of WYLG and schematic diagram of animal experiment process. (a) Blank control group positive/negative total ion flow diagram. (b) WYLG particle treatment group positive/negative total ion flow diagram. (c) Animal experimental protocol.

**Figure:**
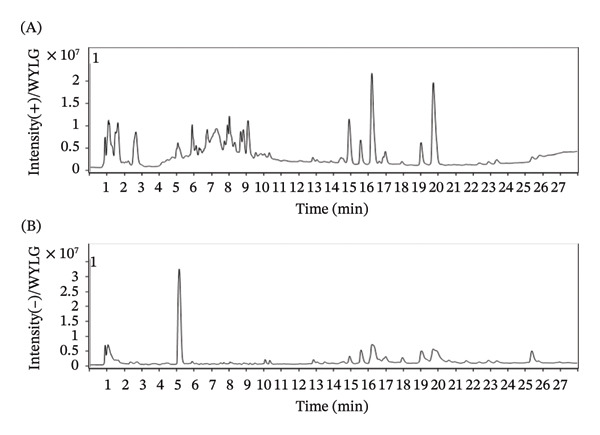
(a)

**Figure:**
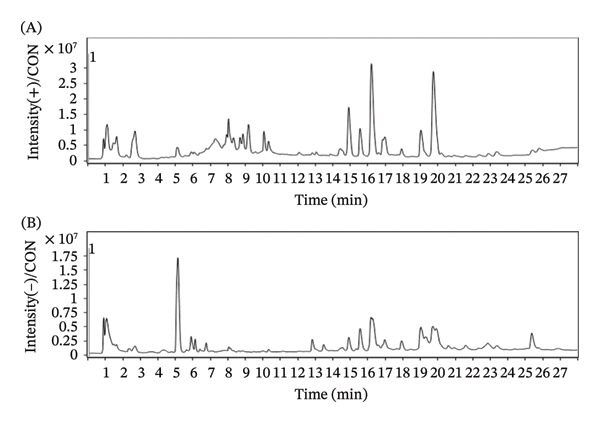
(b)

**Figure:**
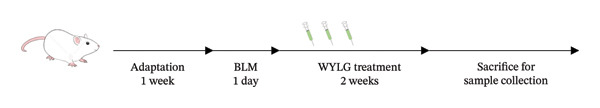
(c)

**Table 1:** The active ingredient of WYLG.

NO.	tR/min	Name	Molecular formula	Ion type	m/z	MS/MS	Source
1	0.975	Caffeic acid	C_9_H_8_O_4_	[M − H]^−^	179.0554	135.0481, 96.9598	*Radix Salviae*
2	1.120	Citric acid	C_6_H_8_O_7_	[M − H]^−^	191.0196	129.0194, 111.0079, 87.0080	*Hedysarum Multijugum Maxim*
3	1.136	Calycosin	C_16_H_12_O5	[M − H]^−^	283.0672	283.0676, 69.0341	*Hedysarum Multijugum Maxim*
4	1.146	L‐isoleucine	C_6_H_13_NO_2_	[M+H]^+^	132.1014	86.0961, 69.0692, 56.0480	*Hedysarum Multijugum Maxim*
5	1.146	Methylmalonic acid	C_4_H_6_O_4_	[M − H]^−^	117.0192	99.0085, 73.0295	*Hedysarum Multijugum Maxim*
6	1.420	Pantothenic acid	C_9_H_17_NO_5_	[M − H]^−^	218.1033	146.0823, 88.0405, 71.0517	*Hedysarum Multijugum Maxim*
7	2.002	Vanillin acid	C_8_H_8_O4	[M − H]^−^	167.0313	167.0323, 123.0414	*Radix Salviae*
8	2.107	Adenine	C_5_H_5_N_5_	[M+H]^+^	136.0621	119.0345, 92.0232	*Hedysarum Multijugum Maxim*
9	2.181	Dihydroxyphenylpropionic acid	C_18_H_16_O_5_	[M − H]^−^	181.0506	181.0466, 137.0184	*Radix Salviae*
10	2.566	L‐tryptophan	C_11_H_12_N_2_O_2_	[M − H]^−^	203.0826	159.0919, 142.0648, 116.0507	*Hedysarum Multijugum Maxim*
11	3.795	Tanshinone IIA	C_19_H_18_O_3_	[M+H]^+^	295.1329	295.1318, 277.1103, 249.1271	*Radix Salviae*
12	3.926	Tanshinol	C_9_H_10_O_5_	[M − H]^−^	197.0428	123.0465, 72.9912	*Radix Salviae*
13	4.102	Ferulic acid	C_10_H_10_O_4_	[M − H]^−^	193.0501	178.0254, 134.0379	*Hedysarum Multijugum Maxim*
14	6.920	Genistin	C_21_H_20_O_10_	[M − H]^−^	431.1000	431.0932, 327.0565, 59.0147	*Hedysarum Multijugum Maxim*
15	7.118	Azelaic acid	C_9_H_16_O_4_	[M − H]^−^	187.0977	169.0898, 143.1078, 125.0961	*Hedysarum Multijugum Maxim*
16	8.381	Cryptotanshinone	C_19_H_20_O_3_	[M − H]^−^	295.1324	295.1311, 83.0499, 55.0185	*Radix Salviae*
17	9.102	Dodecanedioic acid	C_12_H_22_O_4_	[M − H]^−^	229.1447	211.1337, 167.1425	*Hedysarum Multijugum Maxim*
18	23.300	Linoleic acid	C_18_H_32_O_2_	[M+H]^+^	281.2479	263.2372, 71.0856, 57.0693	*Hedysarum Multijugum Maxim*

### 3.2. WYLG Alleviates BLM‐Induced Early Pulmonary Fibrosis in Rats

HE staining showed that compared with the CON group, the BLM group exhibited significant inflammatory cell infiltration, alveolar structure destruction, and interstitial thickening. In contrast, the WYLG treatment groups, especially the WYLG‐H group, showed a significant reduction in inflammatory cell infiltration and improvement in alveolar structure (Figure 2(a)(A)). Semiquantitative scoring of HE staining confirmed that WYLG treatment significantly reduced lung tissue inflammation (Figure 2(a)(B)). Masson staining results showed that the BLM group had a significant increase in collagen deposition compared with the CON group, while WYLG treatment significantly reduced collagen deposition, and the collagen volume fraction was significantly lower than that in the BLM group (Figure 2(b)). These results indicate that WTLG can alleviate BLM‐induced pulmonary fibrosis in rats.

Figure 2Effect of WYLG on BLM‐induced early pulmonary fibrosis in rats. (a) HE staining of rat lung tissues at five time points (1, 3, 5, 7, and 14 days post‐BLM induction). (b) Masson staining of rat lung tissues at five time points (1, 3, 5, 7, and 14 days post‐BLM induction). The results of HE and Masson staining revealed that BLM induction induced pulmonary inflammatory responses in rats, and by day 5, pulmonary interstitial fibrosis and collagen deposition were observed to initiate. Notably, WYLG effectively alleviated these pathological changes (scale bar = 2.5 μm in A and B; ^∗#^ *p* < 0.05, ^∗∗^/^##^ *p* < 0.01; ^∗^ vs. CON group, ^#^ vs. MOD group).

**Figure:**
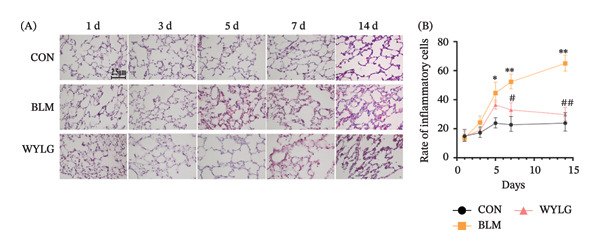
(a)

**Figure:**
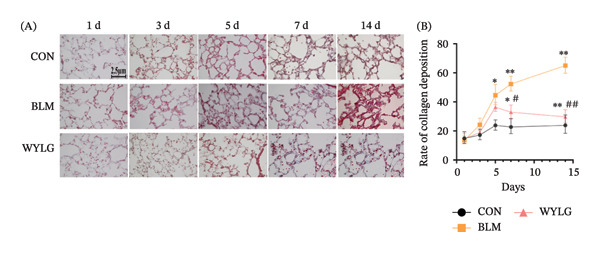
(b)

### 3.3. WYLG Reduces Inflammatory Infiltration and Regulates EMT‐Related Protein Expression in Rat Lung Tissues

To verify the antifibrotic effect of WYLG on BLM‐induced pulmonary fibrosis, HE and Masson staining were performed to observe the pathological changes of lung tissues in rats treated with different doses of WYLG. Compared with the BLM‐induced model group, the high‐dose WYLG group exhibited the least inflammatory cell infiltration, the least severe pulmonary interstitial fibrosis, and the lowest collagen deposition in lung tissues, indicating the most significant therapeutic efficacy (Figures 3(a) and 3(b)). WB analysis of Collagen I expression further confirmed the therapeutic efficacy of WYLG in inflammatory infiltration and fibrosis in lung tissue induced by BLM (Figure 3(c)). The level of IL‐6 in the rat lung tissues was measured to assess the degree of inflammatory infiltration. Compared with the control group, elevated levels of IL‐6 in the BLM group were dose‐dependently downregulated by WYLG administration, indicating that WYLG can attenuate inflammatory infiltration in the lung tissues of rats with BLM‐induced early pulmonary fibrosis (Figure 4(a)). Similarly, the expression of α‐SMA, a canonical marker of myofibroblast activation (a key pathological event in pulmonary fibrosis), was significantly downregulated in the middle‐ and high‐dose WYLG groups compared with the BLM‐induced model group. This finding indicates that WYLG can inhibit myofibroblast activation in the lung tissue of BLM‐induced pulmonary fibrosis rats (Figure 4(b)). E‐cadherin (epithelial cadherin), a key cell‐cell adhesion molecule, is critical for maintaining epithelial integrity. Its downregulation compromises cell–cell adhesion, leading to epithelial cell dissociation and enhanced potential for interstitial transition—hallmarks of EMT in pulmonary fibrosis. Compared with the BLM‐induced model group, the E‐cadherin expression level in the high‐dose WYLG group was significantly elevated, indicating that WYLG contributes to preserving epithelial cell integrity and inhibiting EMT, thereby exerting an antifibrotic effect (Figure 4(c)).

Figure 3WYLG alleviates inflammatory infiltration and fibrosis of lung tissue in rats with early pulmonary fibrosis. (a) HE staining and statistical chart of rat lung tissue after 14 days of WYLG treatment. (b) Masson staining and statistical chart of rat lung tissue after 14 days of WYLG treatment, reflecting the alleviating effects of WYLG on BLM‐induced pulmonary fibrosis and inflammation in rats through staining. (c) (A) The level of Collagen I protein in lung tissue reflects the improvement of WYLG on BLM‐induced collagen deposition in rats. (c) (B) Densitometric quantification of Collagen I versus GAPDH for experiments in (c) (A). The results of the staining experiment were validated by detecting the expression levels of Collagen I protein. The arrow indicates representative cells of pathological changes (scale bar = 10 μm in A and B; ^∗^/^#^/^^^ *p* < 0.05, ^∗∗^/^##^ *p* < 0.01, ^∗∗∗^/^###^ *p* < 0.001, ^∗∗∗∗^/^####^ *p* < 0.0001; ^∗^ vs. CON group, ^#^ vs. MOD group, ^^^ vs. WYLG‐L group).

**Figure:**
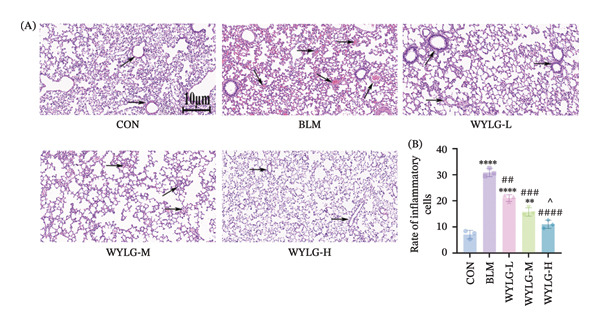
(a)

**Figure:**
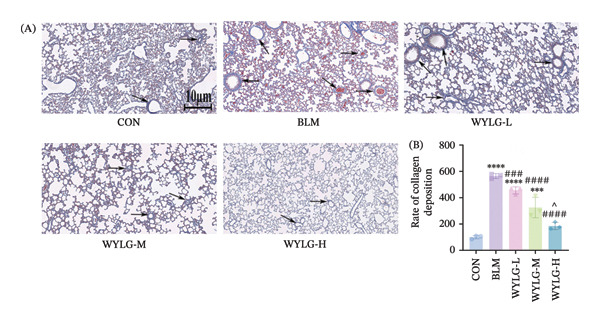
(b)

**Figure:**
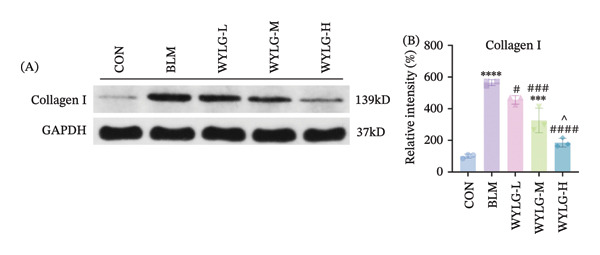
(c)

Figure 4Effects of WYLG on BLM‐induced inflammatory response, cytoskeletal damage, and TGF‐β1/Smad pathway activation in rat lung tissues A, B, C: The levels of IL‐6 (a), α‐SMA (b), and E‐cad (c) in lung tissue were detected by ELISA to reflecting the inflammatory response and the extent of cytoskeletal damage induced by BLM in rats. (d) (A) The level of TGF‐β1/Smad signaling pathway protein in the lung tissue by WB to reflect the treatment of early pulmonary fibrosis by WYLG. (d) (B) Densitometric quantification of TGF‐β, Smad7, Smad2, and p‐Smad2 versus GAPDH for experiments in (d) (A). Meanwhile, the protein levels of p‐Smad2 should be normalized to those of Smad2 for quantitative analysis (^∗^/^#^/^^^ *p* < 0.05, ^∗∗^/^##^/^^^^ *p* < 0.01, ^###^ *p* < 0.001, ^∗∗∗∗^/^####^ *p* < 0.0001; ^∗^vs. CON group, ^#^vs. MOD group, ^^^vs. WYLG‐L group).

**Figure:**
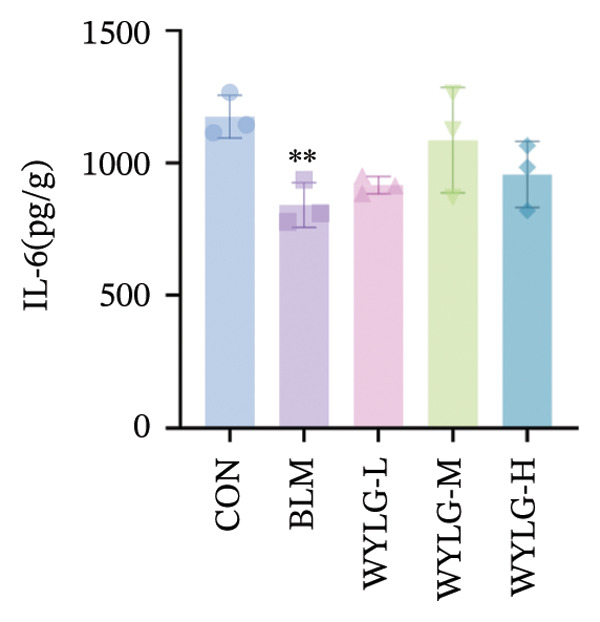
(a)

**Figure:**
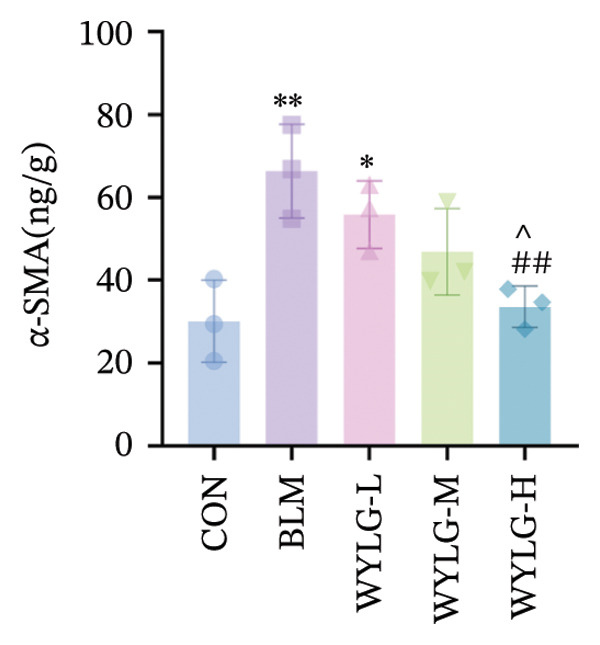
(b)

**Figure:**
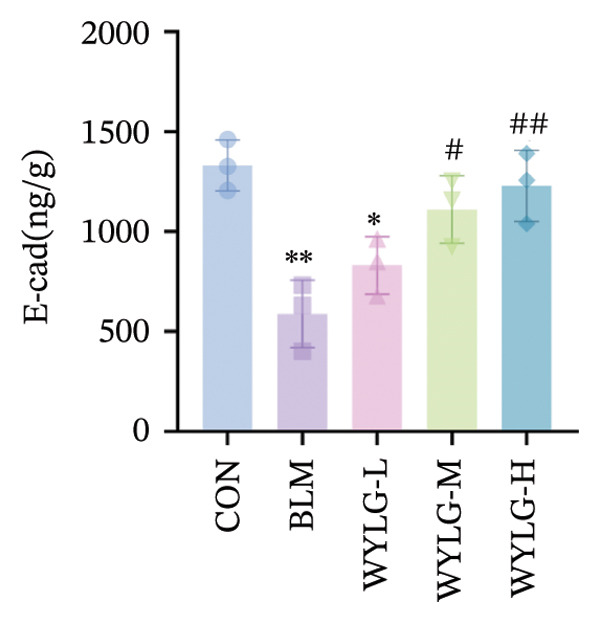
(c)

**Figure:**
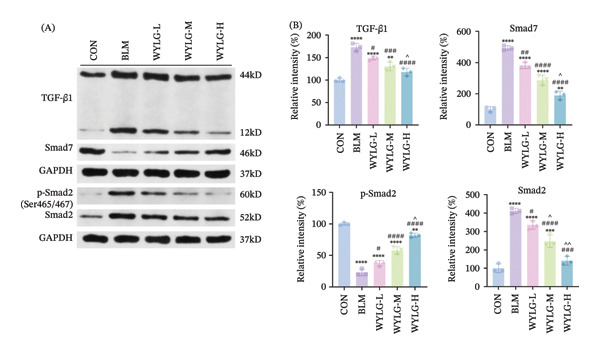
(d)

### 3.4. WYLG Inhibits the TGF‐β1/Smad Signaling Pathway in BLM‐Induced Pulmonary Fibrosis Rats

WB analysis was used to detect the expression of TGF‐β1/Smad pathway‐related proteins in rat lung tissues. The results showed that, compared to the CON group, the expression levels of TGF‐β1 and p‐Smad2/Smad2 in the BLM group were significantly increased, and the expression level of Smad7 was significantly decreased (Figure 4(d)). In contrast, WYLG treatment significantly reduced the expression levels of TGF‐β1 and p‐Smad2/Smad2 and increased the expression level of Smad7, with the most significant effect in the WYLG‐H group. These findings indicate that WYLG can inhibit the activation of the TGF‐β1/Smad signaling pathway in BLM‐induced pulmonary fibrosis rats.

### 3.5. WYLG Ameliorates TGF‐β1‐Induced Fibrosis in RPFs via Regulating the TGF‐β1/Smad Signaling Pathway

To further explore the underlying mechanism of WYLG in treating early pulmonary fibrosis in vitro, we successfully established a TGF‐β1‐induced fibrotic cell model by treating RPFs with TGF‐β1. Subsequently, the model cells were treated with WYLG‐containing drug serum (10% v/v). IF staining confirmed the characteristics of the cell line (Figure 5(a)). CCK‐8 assay results showed that TGF‐β1 at a concentration of 10 ng/mL significantly promoted RPFs proliferation, and this concentration was chosen as the optimal concentration for inducing cell fibrosis (Figure 5(b)). WYLG‐containing serum at a concentration of 10% significantly inhibited TGF‐β1‐induced RPFs proliferation without obvious cytotoxicity, so this concentration was selected as the optimal concentration for subsequent experiments (Figure 5(c)).

Figure 5RPFs characterization, concentration optimization, and cell proliferation analysis of WYLG‐containing serum on TGF‐β1‐induced RPFs. (a) Immunofluorescence identification of RPFs (scale bar = 5 μm). (b) CCK8 to determine the optimal concentration of TGF‐β1. (c) CCK8 to determine the most suitable concentration of drug‐containing serum. (d) (A) Flow cytometry to detect the cell cycle to reflect the effect of serum containing WYLG on TGF‐β1‐induced cell proliferation. (d) (B) Statistical chart of the proportion of cell proliferation at various stages (^∗^ *p* < 0.05, ^∗∗^ *p* < 0.01, ^∗∗∗^ *p* < 0.001, ^∗∗∗∗^ *p* < 0.0001; ^∗^vs. CON group).

**Figure:**
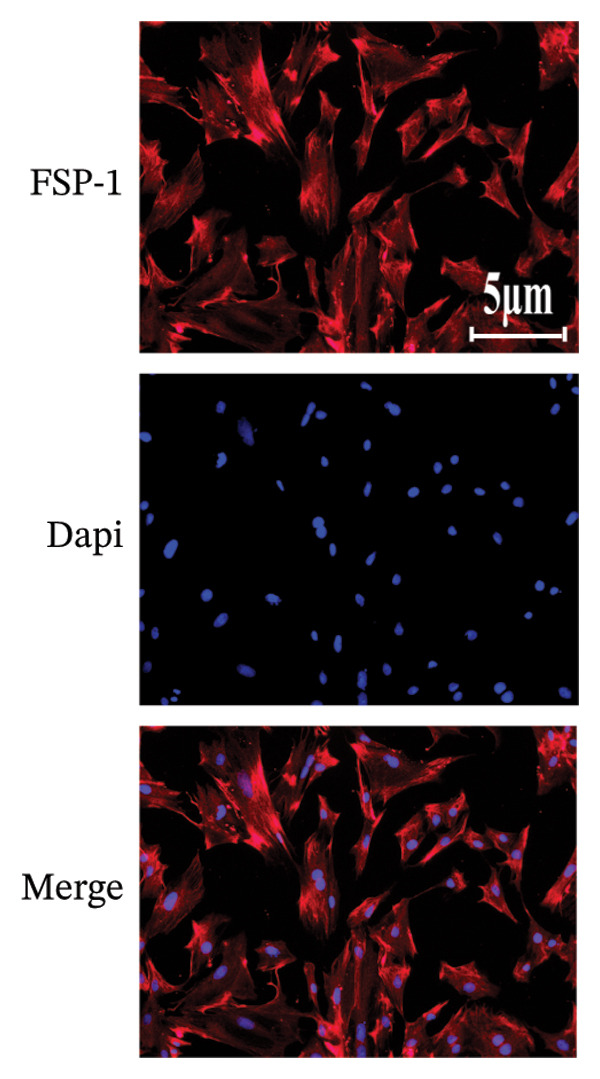
(a)

**Figure:**
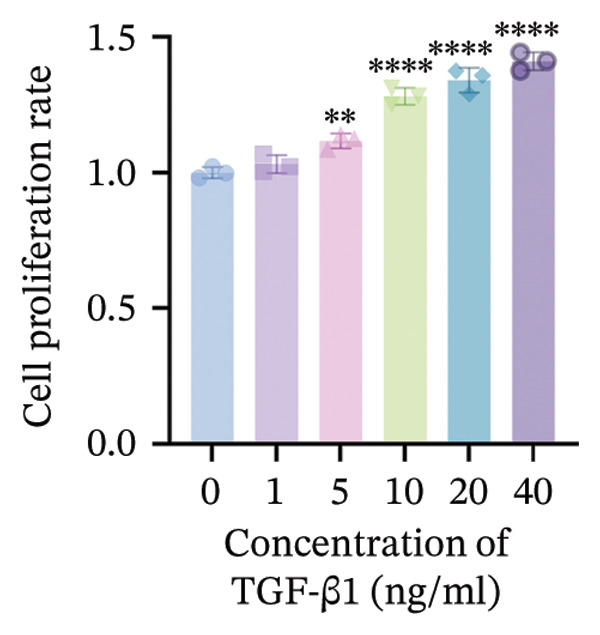
(b)

**Figure:**
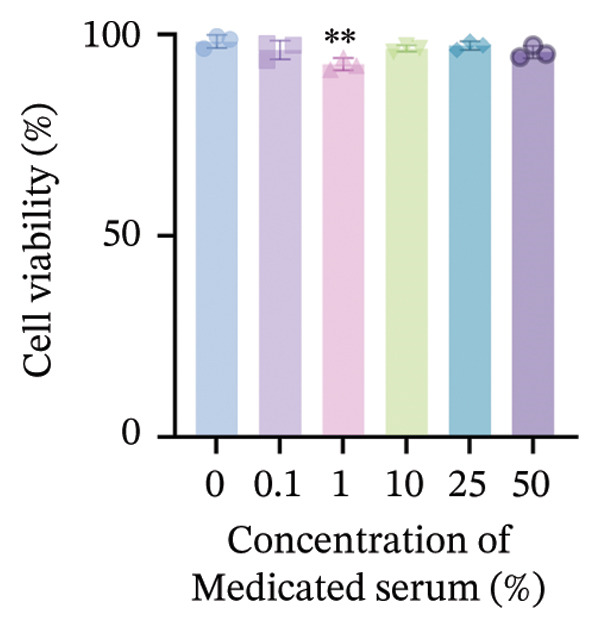
(c)

**Figure:**
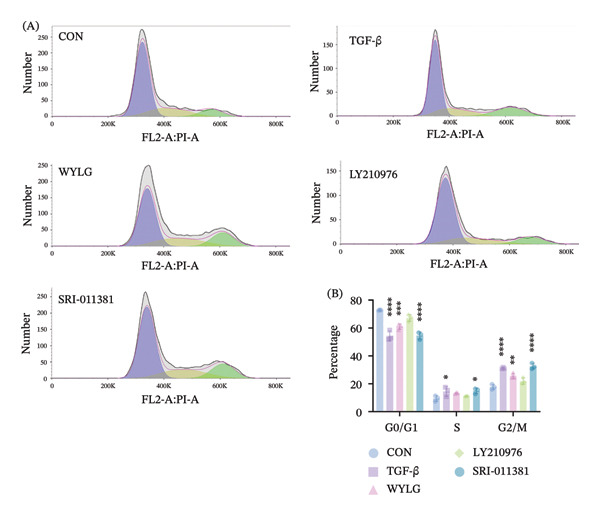
(d)

Flow cytometry results showed (Figure 5(d)) that compared with the CON group, the proportion of G0/G1 phase cells in the TGF‐β1 group was significantly decreased, and the proportion of G2/M phase cells was significantly increased. After WYLG treatment, the proportion of G0/G1 phase cells was significantly increased, and the proportion of G2/M phase cells was significantly decreased, indicating that WYLG can inhibit TGF‐β1‐induced RPFs proliferation. Scratch assay results showed that compared with the TGF‐β1 group, the migration rate of RPFs in the TGF‐β1 + WYLG group was significantly decreased (Figure 6(a)), indicating that WYLG can inhibit TGF‐β1‐induced RPFs migration.

Figure 6RPFs migration, Collagen I, and E‐cadherin detection following WYLG‐containing serum treatment. (a) (A) Cell scratch assay migration area chart (scale bar = 100 μm). (a) (B) Cell scratch assay migration rate statistical chart. (b) (A) Level expression of Collagen I protein in cells. (b) (B) Densitometric quantification of Collagen I versus GAPDH for experiments in (b) (A). (c) Detection of E‐cadherin content in cells by immunofluorescence (^^^ *p* < 0.05, ^∗∗/##^ *p* < 0.01, ^∗∗∗/###^ *p* < 0.001, ^∗∗∗∗^ *p* < 0.0001; ^∗^vs. CON group, ^#^vs. MOD group, ^^^vs. WYLG group).

**Figure:**
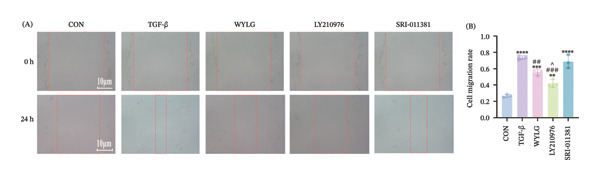
(a)

**Figure:**
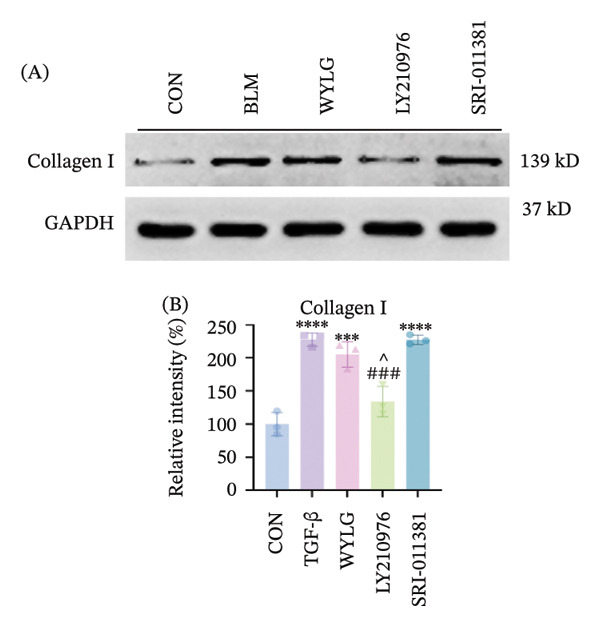
(b)

**Figure:**
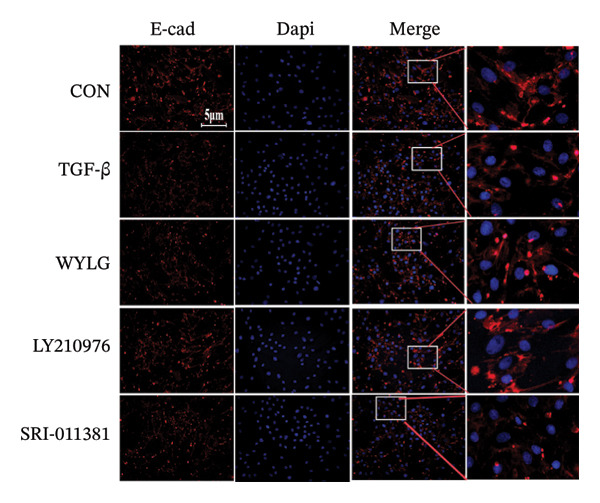
(c)

**Figure 7:**
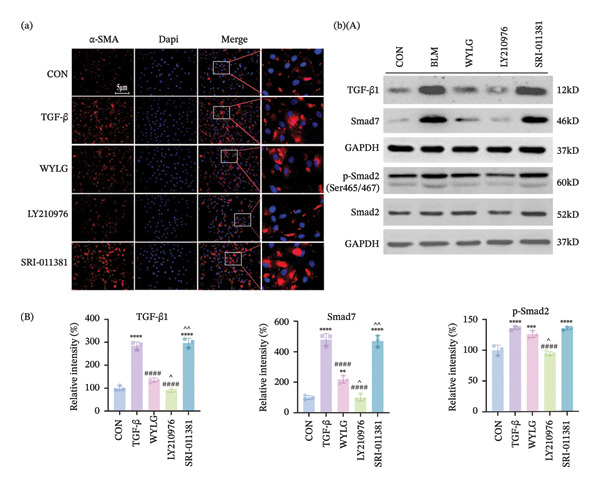
WYLG ameliorates TGF‐β1‐induced fibrotic changes in RPFs through the TGF‐β1/Smad signaling pathway. (a) Detection of α‐SMA content in cells by immunofluorescence (scale bar = 5 μm). (b) (A) The level of TGF‐β1/Smad signaling pathway protein in RPFs by WB to reflect the treatment of pulmonary fibrosis by WYLG. (b) (B) Densitometric quantification of TGF‐β1, Smad7, p‐Smad2 versus GAPDH for experiments in (b) (A). Meanwhile, the protein levels of p‐Smad2 should be normalized to those of Smad2 for quantitative analysis (^^^ *p* < 0.05, ^∗∗/^^^ *p* < 0.01, ^∗∗∗^ *p* < 0.001, ^∗∗∗∗/####^ *p* < 0.0001; ^∗^vs. CON group, ^#^vs. MOD group, ^^^vs. WYLG group).

Additionally, WB analysis indicated that WYLG effectively alleviated TGF‐β1‐induced fibrosis in RPFs by reducing Collagen I protein levels—a finding further validated by comparative analysis using specific inhibitors and agonist of the TGF‐β1/Smad signaling pathway (Figure 6(b)). Finally, we explored whether WYLG improved the fibrotic phenotype in RPFs through the TGF‐β1/Smad molecular signaling pathway. IF results showed that compared with the CON group, the fluorescence intensity of E‐cadherin in the TGF‐β1 group was significantly decreased, and the fluorescence intensity of α‐SMA was significantly increased. WYLG treatment reversed these changes, resulting in increased E‐cadherin fluorescence intensity and decreased α‐SMA fluorescence intensity (Figures 6(c) and 7(a)). WB results showed that compared with the CON group, the expression levels of TGF‐β1 and p‐Smad2/Smad2 in the TGF‐β1 group were significantly increased, and the expression level of Smad7 was significantly decreased. WYLG treatment significantly reversed these changes, similar to the effect of the TGF‐β1 inhibitor LY2109761 (Figure 7(b)). Furthermore, the addition of the TGF‐β1/Smad pathway agonist SRI‐011381 reversed the inhibitory effect of WYLG on the TGF‐β1/Smad pathway, as evidenced by increased expression levels of TGF‐β1 and p‐Smad2/Smad2 and decreased expression level of Smad7 compared with the TGF‐β1 + WYLG group (Figure 7(b)). These results confirm that WYLG can inhibit the TGF‐β1/Smad signaling pathway in RPF cells, thereby exerting an antifibrotic effect.

## 4. Discussion

BLM is one of the most commonly used drugs for inducing lung fibrosis in animal models [22]. Following a single administration of a fibrotic inducer (e.g., BLM), an acute inflammatory response may persist for several days, with its duration depending on the route of administration and dosage. If left unresolved, this acute inflammation ultimately progresses to lung fibrosis. In our preliminary experiments, establishing the BLM‐induced fibrotic model via intratracheal instillation of BLM, the rats exhibited an immediate pulmonary inflammatory response. Local interstitial collagen deposition began on the third day and progressed to extensive collagen deposition by the fifth day. Therefore, in the absence of intervention, the fibrotic process will gradually worsen, making it an ideal stage for studying lung fibrosis. According to “*Plain Questions: Discussion on Bi Syndrome*” in the *Yellow Emperor’s Classic of Internal Medicine*, lung fibrosis is categorized as “lung atrophy,” with the pathogenesis described as “qi deficiency and blood stasis.” The basic treatment principles in TCM are to strengthen the body resistance to eliminate pathogens and promote blood circulation to remove blood stasis [23]. The renowned prescription is Xue’s Five‐Leaf Reed Root Decoction. WYLG is derived from this prescription by removing loquat leaf and lotus leaf, and adding honey‐fried astragalus root, white atractylodes rhizome, raw licorice root, red salvia root, and glandular adenophora root. Among these ingredients, mentha, lotus leaf, agastache, and reed root have light and agile medicinal properties, which can eliminate dampness in the triple energizer; honey‐fried astragalus root and white atractylodes rhizome tonify the spleen and stomach qi; glandular adenophora root nourishes yin and moistens the lungs, while red salvia root promotes blood circulation to remove blood stasis.

In this study, our animal experiments showed that WYLG can alleviate BLM‐induced pulmonary fibrosis in rats, as evidenced by reduced inflammatory cell infiltration, decreased collagen deposition, and improved alveolar structure. Mechanistically, WYLG was found to regulate the expression of EMT‐related proteins, including downregulating α‐SMA and upregulating E‐cadherin, and inhibit the activation of the TGF‐β1/Smad signaling pathway by reducing the expression of TGF‐β1 and p‐Smad2/Smad2 and increasing the expression of Smad7.

To further clarify the mechanism of action of WYLG, cellular experiments were conducted in this study. TGF‐β1 was used to induce pulmonary fibrosis in RPFs to simulate the in vitro fibrotic process. The results showed that WYLG‐containing serum could inhibit the proliferation and migration of TGF‐β1‐induced RPFs, regulate the expression of EMT‐related proteins, and suppress the activation of the TGF‐β1/Smad signaling pathway. Moreover, the addition of the TGF‐β1/Smad pathway agonist SRI‐011381 reversed the antifibrotic effect of WYLG, further confirming that WYLG exerts its antifibrotic effect through the TGF‐β1/Smad signaling pathway. Meanwhile, the antifibrotic effect of WYLG exhibited a similar dose‐dependent pattern in both animal and cellular experiments. This indicates that the mechanism is not an “incidental phenomenon” in animals but a direct effect on lung fibroblasts—the key effector cells in fibrosis. Consistent results from cellular and animal experiments provide reliable evidence for the mechanism of WYLG in the treatment of pulmonary fibrosis. In addition, the cellular experiments yielded three key novel findings. First, the “functional regulatory details” of WYLG on fibrotic cells were clarified. While animal experiments only observed overall improvements in lung pathology, cellular experiments further revealed that WYLG can directly inhibit the abnormal proliferation (evidenced by increased proportion of G0/G1 phase cells and blocked cell division) and migration ability (decreased wound healing rate) of lung fibroblasts. These are critical steps in “fibroblast dissemination and excessive collagen deposition” during pulmonary fibrosis progression, suggesting that WYLG can not only “reduce existing fibrosis” but also prevent the “dissemination and exacerbation” of fibrosis. Second, pathway specificity was confirmed via “agonist rescue experiments”: Animal experiments established the association between WYLG and the TGF‐β1/Smad pathway, while cell experiments used TGF‐β1/Smad pathway agonists (SRI‐011381) and inhibitors (LY2109761) to confirm the role of WYLG from both positive and negative perspectives. The results showed that the agonist abrogated the antifibrotic effect of WYLG, characterized by restored pathway activity and re‐emergence of abnormal EMT phenotypes. This further confirms that WYLG exerts its anti‐fibrotic effect primarily through regulating this pathway rather than alternative bypass pathways. Third, a direct “drug concentration–cell effect” relationship was established: Cellular experiments determined the optimal conditions (10 ng/mL TGF‐β1 for induction and 10% WYLG‐containing serum for intervention) using the CCK‐8 assay, defining the effective concentration range of WYLG at the cellular level. This provides key data for potential future clinical translation.

Modern medical research indicates that EMT is directly related to fibrosis in many organs and is associated with the regulation of multiple signaling pathways such as Ras, Rho, Src, and Smads [24, 25]. TGF‐β1, a subtype of TGF‐β, is widely regarded as one of the important fibrotic factors in the mechanism of EMT‐mediated pulmonary fibrosis [26]. TGF‐β activates TGF‐β1 and TGF‐β2, which in turn activate downstream Smad phosphorylation [27]. Smad7 serves as a core negative regulator of the TGF‐β1/Smad signaling pathway, exerting a pivotal antifibrotic role during the pathogenesis of pulmonary fibrosis. Notably, our findings have demonstrated that WYLG exerts its antifibrotic effects partially by upregulating Smad7 expression. Research shows Smad7 inhibits TGF‐β1‐mediated pro‐fibrotic signaling and attenuates key pathological processes of pulmonary fibrosis—including myofibroblast activation, excessive deposition of Collagen I, and EMT—thus emerging itself as a critical therapeutic target for pulmonary fibrosis. Mechanistically, this antifibrotic role may involve the formation of a complex between Smad7 and phosphorylated Smad2/Smad3 (p‐Smad2/p‐Smad3)—an interaction that sequesters the latter and blocks their nuclear translocation. By sequestering p‐Smad2/p‐Smad3 and inhibiting their transcriptional activity, Smad7 downregulates the expression of downstream pro‐fibrotic target genes (e.g., α‐SMA and Collagen I), suppresses ECM synthesis, enhances matrix metalloproteinase (MMP)‐mediated ECM degradation, and restrains fibroblast‐to‐myofibroblast differentiation [4, 28–30].

Meanwhile, accumulating evidence indicates that E‐cadherin is an essential component of adherens junctions between epithelial cells and plays a crucial role in preserving epithelial cell integrity and cell polarization [31]. In TGF‐β‐induced EMT, E‐cadherin expression is downregulated, which leads to reduced intercellular adhesion in epithelial cells and their tendency to detach from the basement membrane [32]. Cytoskeletal rearrangement occurs in epithelial cells, and new phenotypic proteins, such as α‐SMA, are expressed. The migration ability of epithelial cells is enhanced, and they gradually transform into fibroblasts and myofibroblasts, secreting large amounts of ECM and synthesizing collagen fibers, which directly leads to the formation of pulmonary fibrosis [33]. WYLG Granule contains multiple active components, and studies have shown that many TCM components in the prescription have antipulmonary fibrosis effects [34]. For example, β‐sitosterol interferes with multiple cell signaling pathways and attenuates pulmonary fibrosis through EMT suppression by inhibiting the TGF‐β1/Snail pathway without significant toxicity. Luteolin has also been found to inhibit TGF‐β1‐induced α‐SMA, type I collagen, and vimentin expression in primary cultured mouse lung fibroblasts [35, 36]. These active components may synergistically contribute to the antifibrotic effect of WYLG [37].

We compared our findings regarding WYLG’s antifibrotic mechanism with those of other TCM prescriptions that target the TGF‐β1/Smad pathway (e.g., Bufei Huoxue Capsule, Xue’s Five‐Leaf Reed Decoction) and highlighted the potential advantages of WYLG’s multi‐component synergistic effect. However, our study is not without limitations. Specifically, we have not fully elucidated the key molecular targets through which WYLG exerts its antifibrotic effects; additionally, the lack of clinical trial data restricts the direct translation of these preclinical findings to clinical practice.

## 5. Conclusion

In conclusion, our study demonstrates that early intervention with WYLG granules can attenuate and reduce the degree of early pulmonary fibrosis in BLM‐induced rats. Mechanistically, WYLG exerts its antifibrotic effect by inhibiting the TGF‐β1/Smad signaling pathway and regulating EMT. The consistent results from in vivo animal experiments and in vitro cell experiments strongly support this mechanism, highlighting the potential of WYLG as a therapeutic strategy for patients with progressive pulmonary fibrosis.

## Author Contributions

Lin Li and Yan‐Jun Duan collaboratively designed this experiment. Jia‐Wei Zeng contributed to data curation, formal analysis, investigation, methodology, and writing–original draft. Lin Li contributed to conceptualization, project administration, resources, supervision, validation, visualization, and writing–review and editing. Yan‐Jun Duan contributed to conceptualization, project administration, resources, supervision, and writing–review.

## Funding

The research received no fundings.

## Ethics Statement

The animal study was approved by the Institutional Review Board of the Ethics Committee of Hubei University of Chinese Medicine.

## Conflicts of Interest

The authors declare no conflicts of interest.

## Data Availability

The data that support the findings of this study are available from the corresponding author upon reasonable request.
